# *Diplazium esculentum* (Retz.) Sw.: Ethnomedicinal, Phytochemical, and Pharmacological Overview of the Himalayan Ferns

**DOI:** 10.1155/2021/1917890

**Published:** 2021-09-02

**Authors:** Prabhakar Semwal, Sakshi Painuli, Kartik M. Painuli, Gizem Antika, Tugba Boyunegmez Tumer, Ashish Thapliyal, William N. Setzer, Miquel Martorell, Mohammed M. Alshehri, Yasaman Taheri, Sevgi Durna Daştan, Seyed Abdulmajid Ayatollahi, Anka Trajkovska Petkoska, Javad Sharifi-Rad, William C. Cho

**Affiliations:** ^1^Department of Biotechnology, Graphic Era University, Dehradun, Uttarakhand, India; ^2^Uttarakhand State Council for Science and Technology, Dehradun, Uttarakhand, India; ^3^Himalayan Environmental Studies and Conservation Organization, Dehradun, Uttarakhand, India; ^4^Uttarakhand Ayurved University, Gurukul Campus (Haridwar), Uttarakhand, India; ^5^Graduate Program of Molecular Biology and Genetics, Institute of Natural and Applied Sciences, CanakkaleOnsekiz Mart University, Canakkale, Turkey; ^6^Department of Molecular Biology and Genetics, Faculty of Arts and Science, CanakkaleOnsekiz Mart University, Canakkale, Turkey; ^7^Department of Chemistry, University of Alabama in Huntsville, Huntsville, AL 35899, USA; ^8^Aromatic Plant Research Center, 230 N 1200 E, Suite 100, Lehi, UT 84043, USA; ^9^Department of Nutrition and Dietetics, Faculty of Pharmacy, And Centre for Healthy Living, University of Concepción, 4070386 Concepción, Chile; ^10^Pharmaceutical Care Department, Ministry of National Guard-Health Affairs, Riyadh, Saudi Arabia; ^11^Phytochemistry Research Center, Shahid Beheshti University of Medical Sciences, Tehran, Iran; ^12^Department of Biology, Faculty of Science, Sivas Cumhuriyet University, 58140 Sivas, Turkey; ^13^Beekeeping Development Application and Research Center, Sivas Cumhuriyet University, 58140 Sivas, Turkey; ^14^Department of Pharmacognosy and Biotechnology, School of Pharmacy, Shahid Beheshti University of Medical Sciences, Tehran, Iran; ^15^H.E.J. Research Institute of Chemistry, International Center for Chemical and Biological Sciences, University of Karachi, Karachi 75270, Pakistan; ^16^Faculty of Technology and Technical Sciences, St. Kliment Ohridski University-Bitola, Dimitar Vlahov, 1400 Veles, North Macedonia; ^17^Department of Clinical Oncology, Queen Elizabeth Hospital, Kowloon, Hong Kong

## Abstract

The genus *Diplazium* (family: Athyriaceae) comprises approximately 350 species of pteridophytes*. Diplazium esculentum* (Retz.) Sw. is an important member of this genus and commonly known as a wild vegetable in the Himalayan and sub-Himalayan communities. According to the literature analysis, *D. esculentum* was traditionally used for the prevention or treatment of several diseases such as diabetes, smallpox, asthma, diarrhea, rheumatism, dysentery, headache, fever, wounds, pain, measles, hypertension, constipation, oligospermia, bone fracture, and glandular swellings. Various extracts of *D. esculentum* were evaluated to elucidate their phytochemical and pharmacological activities. A wide array of pharmacological properties such as antioxidant, antimicrobial, antidiabetic, immunomodulatory, CNS stimulant, and antianaphylactic activities have been recognized in different parts of *D. esculentum*. The review covers a systematic examination of pharmacognosy, phytochemistry, and pharmacological applications of *D. esculentum*, but scientifically, it is not fully assessed regarding complete therapeutic effects, toxicity, and safety in the human body. The published literature on *D. esculentum* and its therapeutic properties were collected from different search engines including Wiley online, PubMed, Springer Link, Scopus, Science Direct, Web of Science, Google Scholar, and ACS publications by using specific terms such as “*Diplazium esculentum,* bioactive compounds, biological activities and health benefits” from 1984 to 2021 (March). Therefore, further studies are required to identify the detailed action mechanism of *D. esculentum in vitro/in vivo*, and also, more studies should focus on conservation, cultivation, and sustainable utilization of the species.

## 1. Introduction

The Himalayan botanicals are well known to produce wide variety of secondary metabolites due to critical climatic conditions [1–4]. These botanicals, including wild plants, have a significant role in food security and socio-economic development of the region [5, 6]. Moreover, these botanicals are locally utilized for food resources, medicines, and other purposes due to the presence of numerous bioactive compounds and high nutritional value [7, 8]. With recent developments in science and technology, the importance of wild plants has been identified as a possible source of nutraceuticals and/or functional foods [9].

Among several high valued functional foods, *Diplazium esculentum* is one of the important species of wild ferns, which is frequently consumed by people living in the hilly areas; it is not growing on much higher altitude. *D. esculentum* (*n* = 41 chromosomes, grade of polyploidy = diploid) is utilized as a traditional vegetable in the Himalayan communities [10]. It is an important member of the genus *Diplazium* which comprises around ~350 species of pteridophytes, mainly distributed in Asia and Oceania [11].

Specifically, *D. esculentum* is distributed through different parts of the globe including Cambodia, China, India, Indonesia, Japan, Malaysia, Papua New Guinea, Pakistan, Philippines, Singapore, Taiwan, Thailand, Vietnam, and Bangladesh. It grows on the banks of rivers and streams, canals, marshy areas, and hills with an altitudinal range up to 2,300 meters [12, 13].

It is locally known by different names such as English: vegetable fern; India: dung-kek, kari-welli-panna-maravara, kasrot, kukari-sag, mairungshai, para-panna-maravara, linguda, kathura; Japan: Kuware-shida; Malay: paku, paku-tanjong; Nepali: paninyuro, piraunli; Papua New Guines: sigogo; Philippines: Pako; Thai: kut-kin; and Bangladesh: Dheki Shak [14].

**Figure 1:**
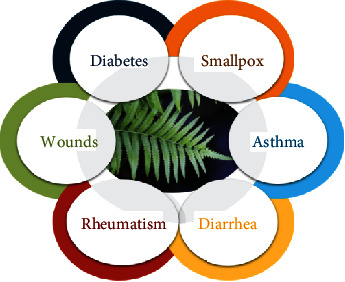
Traditional uses of *Diplazium esculentum*.

Traditionally, *D. esculentum* is being used in the treatment of various ailments (as shown in Figure 1) such as diabetes, smallpox, asthma, diarrhea, rheumatism, dysentery, headache, fever, wounds, pain, measles, high blood pressure, constipation, oligospermia, bone fracture, glandular swellings, and skin-related diseases by the different communities in India and other countries [15–21].

Recently, a few researchers have validated its nutraceutical and pharmacological properties by using *in vitro* and *in vivo* models/methods. For the development of evidence-based medicine, a critical investigation of current knowledge is required regarding ethnopharmacology, chemical composition, biological activities, and possible side effects of the species. Additionally, *D. esculentum* belongs to the least concern category under International Union for Conservation of Nature (IUCN) 2021-1 (https://www.iucnredlist.org/species/194150/8883499) and needs more attention. Therefore, in this manuscript, we reviewed and discuss the recent scientific information conducted so far on *D. esculentum*, which includes its pharmacognosy, phytochemistry, and pharmacology.

## 2. Pharmacognosy

### 2.1. Traditional Uses

Traditionally, *D. esculentum* is one of the most popular vegetables consumed in different parts of the globe, namely, India, Philippines, Nepal, China, Thailand, Indonesia, etc. The literature has revealed that *D. esculentum* is still being used by different communities for the treatment of several diseases including diabetes, smallpox, asthma, diarrhea, rheumatism, dysentery, headache, fever, wounds, pain, measles, and high blood pressure. The detailed information on the traditional uses of the species is summarized in Table 1. Additionally, this species is collected in large amounts and marketed by the rural and tribal communities of India for their livelihood enhancement [22]. The Mishing community of Assam (State of the Indian republic) used it essentially in the religious ceremony of the dead person [23].

### 2.2. Proximate and Mineral Composition

The nutritional value of any food substance can be analyzed by its proximate and mineral composition [24]. Literature-based screening of the proximate composition of *D. esculentum* revealed the presence of lipids, proteins, carbohydrates, vitamins, fiber, etc., while mineral composition possesses the presence of essential micro and macro compounds [25–35]. The comparative analyses of proximate and mineral composition of *D. esculentum* are presented in Tables 2 and 3.

## 3. Bioactive Compounds

Traditionally, botanicals are being widely used to cure various ailments due to the presence of high-valued bioactive compounds [61, 62]. Literature-based screening for bioactive compounds of *D. esculentum* revealed the presence of alkaloids, flavonoids, glycosides, phenolic, tannins, terpenoids, steroids, carbohydrates, fats, and oils in different solvent systems [2, 35, 46, 50, 60, 63–69].

**Figure 2:**
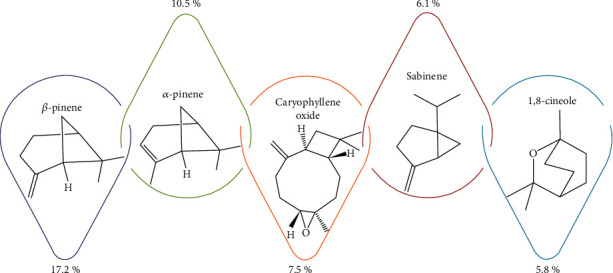
Main essential oil components of *Diplazium esculentum* [16].

In the study of Essien and coworkers [16], the chemical composition of essential oil isolated from *D. esculentum* leaves and the major volatile compounds were identified as *β*-pinene (17.2%), *α*-pinene (10.5%), caryophyllene oxide (7.5%), sabinene (6.1%), and 1,8-cineole (5.8%) (Figure 2). The essential oil of this species was composed of monoterpene hydrocarbons, oxygenated sesquiterpenoids, sesquiterpene hydrocarbons, oxygenated monoterpenoids, and nonterpene derivatives.

**Figure 3:**
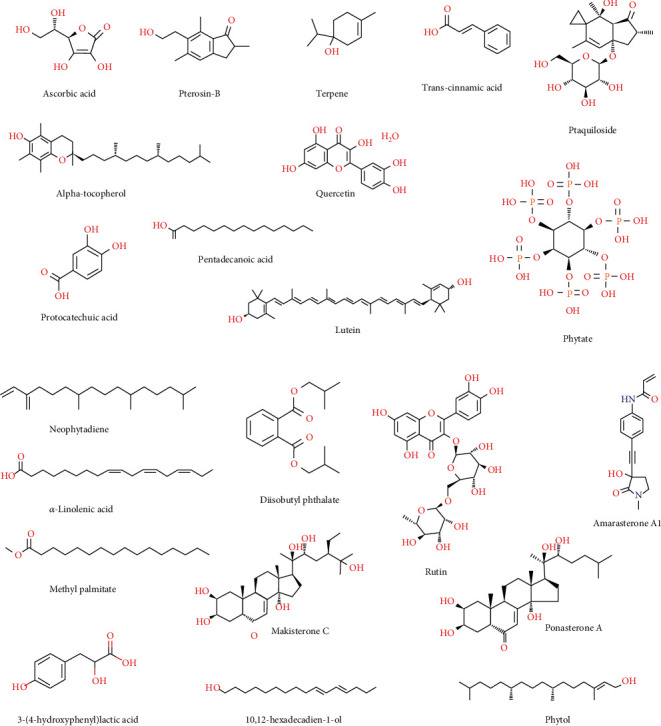
(a) Main nonoil bioactive components of *D. esculentum*. (b) Main nonoil bioactive components of *D. esculentum*.

Few compounds such as ascorbic acid [70], eriodictyol 5-*O*-methyl ether 7-*O*-*β*-D-xylosyigalactoside [71], tannins and phytates [72], *α*-tocopherol [73], quercetin [74, 75], pterosin [75], ptaquiloside [76], terpene, hopan-triterpene lactone [51], and lutein [77] were also isolated from *D. esculentum*. Additionally, four phenolic compounds ((2R)-3-(4′-hydroxyphenyl) lactic acid, trans-cinnamic acid, protocatechuic acid, and rutin) and three ecdysteroids (amarasterone A1, makisterone C, and ponasterone A) were isolated from young fronds of *D. esculentum* collected from Japan [78] while 26 bioactive compounds were identified in the methanolic extracts of young fronds of *D. esculentum* collected from Indian Himalaya [60]. The major compounds present in the species were identified as pentadecanoic acid, *β*-sitosterol, neophytadiene, *α*-linolenic acid, methyl palmitate, diisobutyl phthalate, phytol, and 10,12 hexadecadien-1-ol [60]. These all major compounds are shown in Figures 3(a) and 3(b), respectively.

## 4. Biological Applications

**Figure 4:**
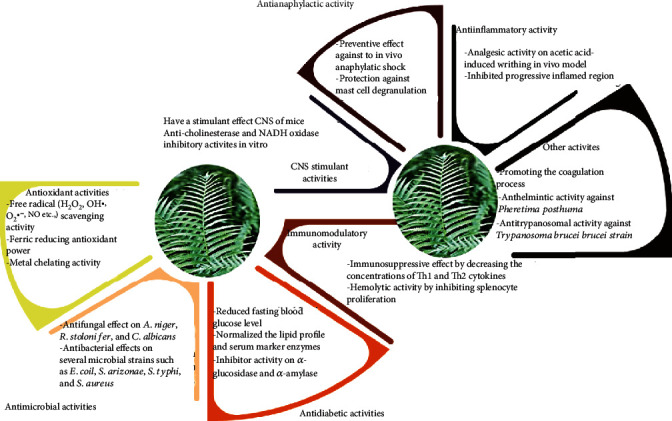
Summary of the proposed biological activities of *Diplazium esculentum*.

Among the functional properties of *D. esculentum*, the antioxidant, anti-inflammatory, antimicrobial, antidiabetic, and immune-modulatory activities can be considered as potentially contributing to the preventive and pharmacological values of this plant species (Figure 4). The following sections reviewed the abovementioned functional biological activities of different *D. esculentum* extracts.

### 4.1. Antioxidant Activities

The botanicals can be considered as safe and cost-effective natural antioxidants capturing free radicals and may help in the prevention and the treatment of different diseases [79, 80]. Recently, a research group from Indonesia reported that the methanolic extract of *D. esculentum* showed a good antioxidant activity with an IC_50_ value of 123.95 ppm according to 2,2-diphenyl-1-picrylhydrazyl (DPPH) radical scavenging activity assay [2].

In an *in vitro* study, the nutritional properties and antioxidant capacity of *D. esculentum* were evaluated on the ethanol extract of the edible parts. The phytochemical analysis indicated that the ethanolic extract possesses significant concentrations of flavonoids (90.6–144.5 mg QE/gm) and tannins (26.8–57.2 mg GAE/gm). Considerable antioxidant activities of *D. esculentum* were revealed using different antioxidant assays including DPPH radical scavenging (IC_50=_146.51 *μ*g/mL), superoxide radical scavenging (IC_50=_111.17 *μ*g/mL), hydroxyl radical scavenging (IC_50=_43.45 *μ*g/mL), and reducing power (IC_50=_76.36 *μ*g/mL) assays.

In another study, the antioxidant activity of *D. esculentum*, extracted by using pressurized hot water extraction (PHWE) method, was reported [81]. The results demonstrated that the optimum condition for the best antioxidant activity of PHWE was at 175°C, 21 min extraction time (2 g dried powder in 50 mL water) in Box-Behnken design. The plant extract showed moderate DPPH scavenging activity (EC_50_ = 1241.14 *μ*g/mL). The hydro-alcoholic extract of *D. esculentum* leaf was evaluated for antioxidant activity using the DPPH and nitric oxide assays [82]. The IC_50_ value of the plant extract for DPPH and NO inhibition activity was found to be 138.8 and 151.9 mg/mL, respectively.

The methanolic extract of *D. esculentum* fronds showed promising antioxidant activity using different assays (DPPH, ABTS, NO, metal chelating, and superoxide scavenging activity) [64]. The IC_50_ values of the plant extract was recorded as 3.8, 4.6, 0.59, and 2.24 mg/mL for DPPH, ABTS, metal chelating, and superoxide scavenging activity, respectively, while nitric oxide, hydroxyl ion, and FRAP assays were recorded as 100-10000 *μ*g/mL, 100-10000 *μ*g/mL, and 0.095-0.121 mM Fe^2+^ equivalent.  Table 4 includes detailed information about previous antioxidant activities.

### 4.2. Antimicrobial Activities

Recently, several pathogenic microorganisms have developed antibiotic resistance, and these antibiotics can have undesirable side effects [92]. Thus, researchers are focusing on botanicals for the development of herbal-based antibiotic substitutes [93].  Table 5 includes antimicrobial studies performed with *D. esculentum*. Antimicrobial activity was considered good (minimum inhibitory concentration (MIC) less than 100 *μ*g/mL), moderate (MIC from 100 to 500 *μ*g/mL), weak (MIC from 500 to 1000 *μ*g/mL), or inactive (MIC over 1000 *μ*g/mL). Inactive results of antimicrobial activities of *D. esculentum* did not included in this study [46, 94].

The areal parts of *D. esculentum* were extracted with ethanol to evaluate the antimicrobial properties by using the disk diffusion method. The crude extract showed considerable antimicrobial activity in terms of minimum inhibitory concentration (MIC) and minimum bactericidal concentration (MBC) value. The MIC value was recorded from a range of 200-800 *μ*g/mL (200 *μ*g/mL (*Bacillus cereus*), 400 *μ*g/mL (*Escherichia coli* and *Aspergillus ochraceus*), and 800 *μ*g/mL (*Bacillus megaterium*)) while MBC from a range of 800 to >800 *μ*g/mL (800 *μ*g/mL (*B. cereus*, *A. ochraceus*) and >800 *μ*g/mL (*B. megaterium*, *E. coli*)), respectively [95].

Different parts (leaves, rhizomes, and roots) of the *D. esculentum* were extracted with aqueous and alcoholic solvents to evaluate the antibacterial activity by using the disk diffusion method. Four bacterial strains, namely, *E. coli*, *Salmonella arizonae*, *Salmonella typhi*, and *Staphylococcus aureus*, were used in this study. The rhizome and root extracts inhibited the growth of microorganisms while leaf extract did not show any inhibition. Additionally, extracts combined with the antibiotic (tetracycline in equal amount) were more potent against bacterial strains than the antibiotic alone [96].

The aerial parts of *D. esculentum* extracts were evaluated for antimicrobial activity by using a colorimetric broth microdilution method. A total six different solvent extracts (hexane, chloroform, ethyl acetate, ethanol, methanol, and distilled water) were used against a series of microbial strains including *S. aureus*, *B. cereus*, *Klebsiella pneumoniae*, *Pseudomonas aeruginosa*, *E. coli*, *Acinetobacter baumannii*, *Candida albicans*, *Candida parapsilosis*, *Issatchenkia orientalis*, *Cryptococcus neoformans*, *Aspergillus brasiliensis*, and *Trichophyton mentagrophytes*. The plant extract only showed a good-moderate antimicrobial activity against *I. orientalis* [97].

The methanolic extract of *D. esculentum* leaves has been evaluated for antibacterial activity by using the disc diffusion method [98]. The plant extract showed slight antibacterial activity (6-10 mm zone of inhibition) against *Salmonella paratyphi*, *Vibrio parahaemolyticus*, *E. coli*, *B. megaterium*, *Shigella dysenteriae*, and *Shigella boydii* among 12 bacterial strains.

The chloroform and methanolic extracts of *D. esculentum* leaves were evaluated for antimicrobial activity by using the disk diffusion method [46]. The plant extracts showed inactive antimicrobial activity against all the microbial strains tested, namely, *K. pneumoniae*, *S. aureus*, *E. coli*, *Salmonella typhimurium*, *Vibrio cholerae*, *Sarcina lutea*, *Bacillus subtilis*, and *Shigella boydii* in terms of MIC (1.6-12.5 mg/mL) value.

The antifungal activity of *D. esculentum* leaves against three fungal strains using the agar diffusion method has been reported [94]. The methanolic extract showed inactive antifungal activity against *Aspergillus niger*, *Rhizopus stolonifer*, and *C. albicans* in terms of MIC (50-100 mg/mL) and minimum fungal inhibition concentration (100-200 mg/mL).

### 4.3. Antidiabetic Activities

Diabetes mellitus is a chronic carbohydrate, fat, and protein metabolism disorder characterized by the increase in blood glucose level due to defect of insulin secretion [99]. The inhibition of *α*-glucosidase and *α*-amylase enzymes, involved in the digestion of carbohydrates, can significantly reduce the postprandial increase of blood glucose and therefore can be an important strategy in the management of blood glucose level in type 2 diabetic and borderline patients. The antidiabetic activity of *D. esculentum* through inhibition of *α*-glucosidase and *α*-amylase enzymes has been reported [69]. The results demonstrated that *D. esculentum* extract exhibited the highest *α*-amylase (92.09%) and *α*-glucosidase (70.01%) inhibitory activities.

The protective effect of a hydro-alcoholic extract of *D. esculentum* on streptozocin- (STZ-) induced diabetes was evaluated [82]. In this study, a total of 30 rats were used and treated with plant extract up to 21 days. After the treatment, it was observed that the plant extract (500 mg/kg) reduced (50.2%) the blood glucose level in STZ-induced diabetic rats. Additionally, a significant reduction was recorded in plant extract-treated rats for lipid profiling (*p* < 0.01), serum marker enzyme activity (*p* < 0.001), necrosis, and regeneration of beta cells. The plant extract showed dose-dependent activity in all the experiments.

### 4.4. Immunomodulatory Activity

The immunosuppressive and hemolytic activities of *D. esculentum* extracts in mouse models have been evaluated [100]. A total of 120 Swiss albino mice (6-8 weeks age) were treated with plant extracts up to 180 days. After this treatment, the plant extract showed significant dose-dependent decreases in body weight, relative spleen weight, number of plaques (formation of antibody secreting cells) formed, hemagglutination antibody titer value, the number of peritoneal macrophages, and the number of cultured splenocytes. The *in vitro* analysis showed significant dose-dependent increases in the percentage inhibition of splenocyte proliferation as well as the percentage of hemolysis. In other words, the treatment with *D. esculentum* may act as an immunosuppressive agent.

The impact of boiled *D. esculentum* on Th1 and Th2 cytokine levels of Swiss albino mice that were treated with different doses of plant extract, daily up to 180 days, has been reported [101]. The outcome of the study demonstrated that the plant extract significantly decreases the concentration of Th1 and Th2 cytokines when compared with controls. In other words, boiled *D. esculentum* extract may affect some of the innate and cell-mediated immune responses by modulating the level of Th1 and Th2 cytokines.

### 4.5. CNS Stimulant Activities

The impact of “Ulam” (a fresh Malaysian vegetable, *D. esculentum*) on cognitive status has been evaluated [102]. In this cross-sectional study, a total of 132 adults were recruited. Socio-demographic information, anthropometric measurements, dietary history, food frequency, and cognitive function were assessed. The average ulam intake by the participants was 15.1 ± 8.2 g/day. The outcome of the study indicated that “pucukpaku” showed protective effects (62.9%) against cognitive decline.

The anticholinesterase and NADH oxidase inhibitory activities of a methanolic extract of *D. esculentum* have been evaluated [83]. Recently, most of the studies reported that the inhibition of anticholinesterase has been shown to be a strategy for the treatment of neurodegenerative disorders. The results of the study demonstrated that the methanolic extract of *D. esculentum* inhibited acetyl-cholinesterase and NADH oxidase in a dose-dependent manner, with IC_50_ values of 272.97 and 265.81 *μ*g/mL.

The CNS stimulant effect of *D. esculentum* in a mouse model using digital acto-photometer has been reported [88]. The plant water extract showed statistically significant (*p* < 0.0001) and dose-dependent activity when compared with control and standard caffeine.

### 4.6. Toxicity Studies (In Vitro and In Vivo)

The methanolic and chloroform extracts of *D. esculentum* were evaluated for their toxicity using brine shrimp lethality bioassay. Both extracts produced dose-dependent increment in percent mortality of brine shrimp nauplii which indicates the presence of toxic compounds in the extracts. The LC_50_ values were recorded as 1.87 *μ*g/mL (chloroform), 1.62 *μ*g/mL (methanol), and 0.66 *μ*g/mL (vincristine sulphate as standard drug) [46]. In another study, the toxicity of methanolic extract of *D. esculentum* using brine shrimp lethality bioassay was reported as significant (LC_50_ = 18.6 *μ*g/mL). [98]. In other study, the cytotoxicity of ethanolic extract of *D. esculentum* was evaluated in different cell lines including breast cancer (MDA-MB-231 and MCF-7), colon cancer (Caco-2), liver cancer (HepG2), and normal liver (Chang liver), and no cytotoxic effect was observed [103].

The systemic toxicity and several pathological effects of *D. esculentum* were evaluated on rabbits and guinea pigs [104]. The study indicated that the plant extract decreased all the pathological functions including growth, body weight, forced motor activity, alterations of blood glucose values, erythrocyte sedimentation rate, mean corpuscular volume, mean corpuscular hemoglobin, total leukocyte count, neutrophil, lymphocyte, and monocyte count, while increased blood SGOT level in both rats and guinea pigs. In other words, the plant extract indicated toxic effects in guinea pigs and rabbits, while rats showed a little adverse effects. Junejo and coworkers reported the nontoxic effects of *D. esculentum* extract on experimental models and recommended as a potential functional food [67].

The toxicological impact of *D. esculentum* on male reproductive functions of Swiss albino mice has been reported [105]. A total of 120 male Swiss albino mice of 6-8 weeks of age were fed orally with 80, 160, and 320 mg/kg b.w. of plant material and treated up to 180 days. After this successful treatment, the boiled plant extract showed significant dose and time-dependent decreases in body weight, absolute and relative testis weight, the relative weight of other organs and their biochemical parameters, percentage of live spermatozoa, fertility, and fecundity in plant extract fed mice. In other words, the main outcome of this study is boiled extracts of *D. esculentum* possess toxic properties that can be slow down the male reproductive functions and may induce infertility.

### 4.7. Antianaphylactic and Mast Cells Stabilizing Activity

*D. esculentum* were extracted with aqueous and ethanolic solvents and evaluated for mast cell stability and antianaphylactic activity. In this study, Swiss albino mice (18-20 g) and Wistar rats (150-170 g) were used. A significant reduction was observed in the number of degranulated mast cells of the plant extracts-treated models (*p* < 0.001). After the administration of both extracts at 250 and 500 mg/kg doses, it showed 72.83%, 76.67%, 69%, and 71.67% intact mast cells. Plant extract demonstrates protective activity against mast cell degranulation. The 500 mg/kg dose of both extracts showed maximum inhibition of the release of myeloperoxidase from lung tissue. Additionally, the plant extract had stabilized the mast cell membrane and decreased the level of nitric oxide in serum and peritoneal fluid [106].

### 4.8. Anti-inflammatory Activity

The ethanolic extract of *D. esculentum* was evaluated for anti-inflammatory activity [107]. A total of 25 male mice were recruited in this experiment and divided into 5 groups. The ethanolic extract indicated anti-inflammatory activity on hind paw oedema in terms of inflamed inhibition percent of 125 mg/kg b.w. (71.72%), 250 mg/kg b.w. (81.49%), and 250 mg/kg b.w. (92.60%) in the treated group. In another study, a considerable analgesic activity of *D. esculentum* was recorded using the acetic acid-induced writhing method in mice [63].

### 4.9. Other Biological Activities

The aqueous and powder extract of *D. esculentum* leaves was evaluated for coagulant activity [108]. The plant extracts combined with polyaluminium chloride showed a synergistic effect for all the measured parameters in Kuala Sepetang Landfill Site (KSLS) leachates. The combination was identified as a high molecular weight polymer, and it acted as an anionic coagulant and was also capable of promoting the coagulation process.

Extracts of the rhizome of *D. esculentum* extracts were evaluated for their anthelmintic activity against *Pheretima posthuma.* The study included three solvents (ethanol, aqueous, and petroleum ether) and three concentrations (10, 25, and 50 mg/mL), and all the extracts demonstrated significant anthelmintic activity in terms of the time of paralysis and time of death. Ethanolic extract showed the highest activity compared to other solvents, and the activity was recorded in dose-dependent patterns [109].

Silver nanoparticles (10-45 nm) were synthesized using the leaf powder of *D. esculentum* [110]. The synthesized nanoparticles were evaluated as a catalyst in the degradation of methylene blue and rhodamine B and also evaluated for the anticoagulation activity. The synthesized Ag NPs showed considerable anticoagulation activity. Besides, prominent photocatalytic activity in the degradation of methylene blue and rhodamine B was also recorded.

The antitrypanosomal activity of *D. esculentum* leaves was evaluated against *Trypanosoma brucei brucei* strain BS221 [111]. In this study, the ethanolic extract was used with seven different concentrations (0.01 to 12.5 *μ*g/mL), and the extract showed significant antitrypanosomal activity with IC_50_ value 4.32 *μ*g/mL and a selectivity index (SI) value > 23 in mammalian cell line (Vero, IC_50_ > 100 *μ*g/mL) when compared with the positive control (pentamidine, IC_50_ = 4.51 ng/mL).

## 5. Concluding Remark and Future Prospective

The present manuscript reports traditional uses, nutraceuticals, pharmacognosy, phytochemistry, and pharmacological studies in *D. esculentum*. The literature survey revealed that *D. esculentum* is one of the most important and popular wild species of ferns in the Himalaya. It is a widely used species in different traditional systems, but the complete chemical composition and active compounds need to be further elucidated and authenticated by bioassay-guided isolation. However, very limited studies are available for this species, not only in terms of chemical characterization but also in terms of pharmacological evaluation as well. Most of the studies are limited to the *in vitro* screening and a few for *in vivo.* Clinical trial studies should be performed to evaluate the safety profile of wild ferns in the human body in terms of antimicrobial activity, antidiabetic activity, anti-inflammatory activity, and immunomodulatory aspects. Apart from this, educating the local people regarding the cultivation, conservation, and sustainable utilization of this plant will help for improving the population size of the species.

## Figures and Tables

**Table 1:** Traditional uses of *D. esculentum* in different regions.

Plant part	Ethno-pharmacological uses	Country	References
Fronds/leaves/areal part/whole plant	Cooked and eaten as a vegetable and in soups to maintain good health.	India, Bangladesh, Thailand	[23, 36–38]
	Tender leaves are cooked with fruit of *Dillenia indica* and fish and taken as vegetable.	Malaysia, India	[39, 40]
	Hairs are removed, boiled with salt and water until water is evaporated then fried and eaten as vegetable.	India	[41–45]
	Used in headache, pain, fever, wounds, dysentery, glandular swellings, diarrhea, measles, toothache, high blood pressure, and various skin infections. Fronds used by pregnant women as protection against difficult childbirth. Leaf paste is used in the wounded place externally for the cure of bone fracture. Used as a laxative. Used as insecticides.	Bangladesh, Nigeria, Indonesia, Nigeria, India, Philippines	[15–17, 19, 46–48]
	The tender frond is cooked without salt and is consumed with rice for 5–10 days for the treatment of diabetes.	India	[20]
	Eaten as highly preferred Koche Sag, Neuro/Niuro vegetable.	Nepal	[49]
	Used as vegetable and pickle.	India, Vietnam, Japan, Indonesia, Philippines	[30, 31, 50–53]

Root	About 20 g of fresh root is boiled in 1 liter of water and reduced to one-fourth of its volume. 3 mL of this decoction along with 2 mL of honey is taken orally on an empty stomach twice a day for 15 days to cure spermatorrhea.	India	[54]
	About 50 g juice obtained from macerated root is fed three times for human dysentery. Macerated root extract is also useful for the cattle dysentery.	Bangladesh	[55]
	About 2-3 spoonsful of root juice are taken for 1/2 days, or 1/½ cup of boiling extract of whole plant is taken thrice daily to treat infections and used as an antidote. The root paste is used externally for the treatment of rheumatism and smallpox. Two pills of pulverized root and honey are taken thrice daily for 2 weeks for the treatment of oligospermia.	Bangladesh	[17]

Rhizome	Decoction of rhizome used as a tonic and also used for the cure of hemoptysis and cough.	India	[39, 56]

**Table 2:** Proximate composition of *Diplazium esculentum* from different regions.

Parameters	Bangladesh (mg/100 g) [34]	Indonesia (%) [29]	India (%) [26]	India (%) [57]	Philippines (%) [33]	Nepal (%) [58]	Indonesia (%) [27]	India (%) [59]	India (%) [60]
Moisture (%)	8.8	—	89.34	92.4	91.82	93.25	90.84	93.1	90.4
Lipid	2.16	—	—	—	—	—	—	—	—
Protein	8.73	6.20-8.30	3.84	31.2	0.87-10.67	0.99	2.23	2.6	8.87
Ash	5.09	1.90-2.11	1.33	16.2	1.42-17.39	1.10	1.38	1.3	—
Total carbohydrate	59.62	—	—	44.3	—	—	—	1.0	18.8
Fiber	15.59	—	5.05	4.6	0.72-9.06	0.99	4.82	—	3.1
Fat	—	0.51-0.68	0.25	8.3	0.28-3.40	0.15	0.04	2.0	2.5
Water level	—	2.70-3.08	—	—	—	—	—	—	—
Vitamin C (mg/100 g)	—	—	21.38	21	—	6.20	—	—	—

**Table 3:** Mineral composition of *Diplazium esculentum* from different regions.

Parameters	Bangladesh (mg/100 g) [32]	Bangladesh (mg/g) [34]	India (mg/100 g) [26]	Indonesia (*μ*g/g) [31]	Malaysia (mg/kg) [28]	India (mg/100 g) [57]	India (mg/g) [25]	Nepal (mg/100 g) [58]	Indonesia (mg/kg) [27]	India (mg/100 g) [59]
N	—	13.97	—	—	—	—		—	—	—
P	48	1.58	—	—	—	—		117	0.09	—
K	—	7.93	—	—	—	914.4		—	0.24	927.4
Ca	9	—	0.66	—	—	192.7		—	0.39	200.5
Mg	11	—	9.56	—	—	0.36	10-12.11	—	0.14	—
Fe	—	—	14.38	15.7	—	11.2	20.2-23.4	1.03	44.6	—
Mn	—	—	11.91	7.03	3.24-22.5	—	0.04-0.38	—	—	—
Na	54	20.21	0.50	—	—	9.5		—	—	8.1
Cu	—	—	13.37	3.99	3.24-24.3	0.32	1.03-1.28	—	4.24	—
Al	—	—	58.5	18.3	—	—	0.10-0.73	—	—	—
As	—	—	14.6	—	—	—	—	—	—	—
Cd	—	—	0.4	—	—	—	—	—	—	—
Hg	—	—	0.07	—	—	—	—	—	—	—
Li	—	—	2.1	—	—	—	—	—	—	—
Ni	—	—	24.5	—	—	—	—	—	—	—
Pb	—	—	0.8	2.46	0.31-3.26	—	—	—	—	—
Cr	—	—	—	0.05	1.19-3.03	—	—	—	—	—

**Table 4:** Previous antioxidant studies in *Diplazium esculentum*.

Plant part used and solvent system	Name of assay	Key results			References
		Plant extracts	Positive control	Antioxidant activity^∗^	
Whole plant, (chloroform, n-butanol, aqueous)	Free radical scavenging (DPPH)	IC_50_ = 287-404 *μ*g/mL	IC_50_ = 17.45 *μ*g/mL	Moderate	[77]
	Radical cation scavenging activity (ABTS^+^)	IC_50_ = 191-273 *μ*g/mL	IC_50_ = 08.44 *μ*g/mL	Moderate	
	Ferric reducing antioxidant power (FRAP)	0.44-0.55 mg/g	—		

Leaves (methanol)	Free radical scavenging (DPPH)	31.35-57.95% inhibition	91.99-97.03% inhibition	Moderate	[57]

Leaves (methanol)	Free radical scavenging (DPPH)	IC_50_ = 402.88 *μ*g/mL	IC_50_ = 324.86 *μ*g/mL	Weak	[83]

Leaves (protein)	Free radical scavenging (DPPH)	IC_50_ = 10.23 mg/mL	—	—	[84]
	Free radical scavenging (DMPD·+)	IC_50_ = 14.67 mg/mL	—	—	
	Radical cation scavenging activity (ABTS^+^)	IC_50_ = 07.95 mg/mL	—	—	

Leaves (not reported)	Free radical scavenging (DPPH)	336-3359 ORAC unit^2^/g	—	—	[50]

Leaves (ethanol, vinegar, acetic acid, aqueous)	Free radical scavenging activity (DPPH)	258-303 *μ*mol TE/100 g	—	—	[85]

Leaves (chloroform, methanol)	Total antioxidant capacities (TAC)	181.94-207.41 mg/g	—	—	[46]
	Free radical scavenging (DPPH)	IC_50_ = 5907-95669 *μ*g/mL	IC_50_ = 13.76 *μ*g/mL	Weak	

Leaves (methanol)	Free radical scavenging activity (DPPH)	IC_50_ = 1.73 mg/mL	—	—	[86]
	Metal chelating activity	52.07 mg/mL	—	—	
	Ferric reducing antioxidant power (FRAP)	2.12 *μ*g/mg	—	—	
	Radical cation scavenging activity (ABTS^+^)	IC_50_ = 0.03 mg/mL	—	—	

Fronds (aqueous, ethanol)	Radical cation scavenging activity (ABTS^+^)	09.60-57.84% inhibition	—	—	[65]
	Hydrogen peroxide scavenging (H_2_O_2_)	15-40% inhibition	50% inhibition	Strong	

Leaves (methanol)	Hydroxyl radical scavenging (OH·)	IC_50_ = 811.00 *μ*g/mL	IC_50_ = 571.00 *μ*g/mL	Weak	[87]
	Superoxide anion scavenging (O_2_·^−^)	IC_50_ = 90.39 *μ*g/mL	IC_50_ = 42.06 *μ*g/mL	Strong	
	Nitric oxide radical scavenging (NO)	IC_50_ = 204.28 *μ*g/mL	IC_50_ = 90.82 *μ*g/mL	Moderate	
	Hydrogen peroxide scavenging (H_2_O_2_)	IC_50_ = 4.17 mg/mL	IC_50_ = 3.24 *μ*g/mL	Strong	
	Peroxynitrite scavenging (ONOO^−^)	IC_50_ = 3.35 mg/mL	IC_50_ = 0.87 *μ*g/mL	Strong	
	Singlet oxygen scavenging (^1^O_2_)	IC_50_ = 278.88 *μ*g/mL	IC_50_ = 46.15 *μ*g/mL	Moderate	
	Hypochlorous acid scavenging (HOCl)	IC_50_ = 338.96 *μ*g/mL	IC_50_ = 235.95 *μ*g/mL	Moderate	
	Iron chelating	IC_50_ = 1.33 mg/mL	IC_50_ = 0.001 *μ*g/mL	Strong	
	Lipid peroxidation inhibition	IC_50_ = 141.67 *μ*g/mL	IC_50_ = 6.76 *μ*g/mL	Moderate	

Leaves (petroleum ether, chloroform, acetone, methanol, aqueous)	Ferric reducing antioxidant power (FRAP)	0.22-7.6 mM/dry weight	—	—	[88]

Leaves (aqueous-methanol, acetone)	Free radical scavenging (DPPH)	IC_50_ = 0.92-3.60 mg dry wt.	—	—	[89]
	Ferric reducing antioxidant power (FRAP)	4.99-8.78 mg/g	—	—	

Leaves (methanol)	Free radical scavenging (DPPH)	EC_50_ = 3353.2 *μ*g/mg	EC_50_ = 322.4 *μ*g/mg	Weak	[90]

Leaves (aqueous)	Free radical scavenging (DPPH)	50 *μ*mol/g	—	—	[91]
	Ferric reducing antioxidant power (FRAP)	100 mol/g	—	—	
	Cupric ions chelation assay	80% inhibition	—	—	

(-): not mentioned in the reference papers; (^∗^): antioxidant activity was considered strong (less than 100 *μ*g/mL), moderate (100 to 500 *μ*g/mL), weak (500 to 1000 *μ*g/mL), or inactive (over 1000 *μ*g/mL) compared with control.

**Table 5:** Antimicrobial activities of *Diplazium esculentum*.

Plant part used and solvent system	Microorganism	Antimicrobial activity	MIC (*μ*g/mL)	MBC or MFC (*μ*g/mL)	Reference
Aerial parts (ethanol)	*Bacillus cereus* *Escherichia coli* *Aspergillus ochraceus* *Bacillus megaterium*	Moderate Moderate Moderate Weak	200 400 400 800	800 >800 800 >800	[95]

Aerial parts (ethanol)	*Staphylococcus aureus* *Bacillus cereus* *Klebsiella pneumoniae* *Pseudomonas aeruginosa* *Candida albicans* *Candida parapsilosis* *Cryptococcus neoformans* *Issatchenkia orientalis*	Moderate-weak Moderate-weak Moderate-weak Weak Weak-inactive Weak-inactive Moderate Good-moderate	310-630 310-630 310-630 630 630-1250 1250-2500 310 80-160	NA 1250 NA NA 2500 NA 310 160	[97]
Aerial parts (hexane)	*Cryptococcus neoformans* *Issatchenkia orientalis*	Moderate Good	310 80	310 160	[97]

Aerial parts (chloroform)	*Staphylococcus aureus* *Bacillus cereus* *Klebsiella pneumoniae* *Pseudomonas aeruginosa* *Trichophyton mentagrophytes* *Candida albicans* *Cryptococcus neoformans* *Issatchenkia orientalis*	Moderate-weak Moderate Moderate-weak Weak-inactive Weak-inactive Weak-inactive Moderate Good	310-630 310 310-630 630-1250 630-1250 630-1250 310 80	NA 630 NA NA 1250 NA 310 160	[97]

Aerial parts (ethyl acetate)	*Pseudomonas aeruginosa* *Cryptococcus neoformans* *Issatchenkia orientalis*	Weak Moderate Moderate	630 310 160	NA 310 310	[97]

Aerial parts (methanol)	*Staphylococcus aureus* *Bacillus cereus* *Klebsiella pneumoniae* *Pseudomonas aeruginosa* *Cryptococcus neoformans* *Issatchenkia orientalis*	Weak-inactive Weak-inactive Weak-inactive Weak-inactive Moderate Moderate	630-1250 630-1250 630-1250 630-1250 310 160	NA 2500 NA NA 310 310	[97]

Aerial parts (aqueous)	*Pseudomonas aeruginosa* *Cryptococcus neoformans* *Issatchenkia orientalis*	Weak-inactive Moderate Moderate	630-1250 160-310 160	NA 310 310	[97]

Leaves, rhizomes, and roots (aqueous and alcoholic)	*Escherichia coli* *Salmonella arizonae* *Salmonella typhi* *Staphylococcus aureus*		-^∗^ -^∗^ -^∗^ -^∗^		[96]

Leaves (methanol)	*Salmonella paratyphi* *Vibrio parahemolyticus* *Escherichia coli* *Bacillus megaterium* *Shigella dysenteriae* *Shigella boydii*		-^∗^ -^∗^ -^∗^ -^∗^ -^∗^ -^∗^		[98]

MBC: minimum bactericidal concentration; MIC: minimum inhibitory concentration; MFC: minimum fungal concentration; NA: no activity; “-”: not tested. Antimicrobial activity was considered good (MIC less than 100 *μ*g/mL), moderate (MIC from 100 to 500 *μ*g/mL), and weak (MIC from 500 to 1000 *μ*g/mL). ^∗^Only zone inhibition test was performed.

## Data Availability

The data used to support the findings of this study are available from the corresponding author upon request.
